# Development and validation of the EDIT weight stigma reduction checklist

**DOI:** 10.1038/s41366-026-02045-y

**Published:** 2026-03-24

**Authors:** Xochitl de la Piedad Garcia, Kelly Cooper, Isabelle R. Jardine, Angela S. Alberga, Katherine E. Darling, Andrew J. Hill, Erica M. Howes, Alaina P. Vidmar, Jacqlyn Yourell, Natalie B. Lister, Hiba Jebeile

**Affiliations:** 1 School of Behavioural and Health Sciences, Australian Catholic University, Melbourne, VIC Australia; 2 The Weight Issues Network, Sydney, NSW Australia; 3 Sydney Medical School, The University of Sydney, Westmead, NSW Australia; 4 Department of Health, Kinesiology, and Applied Physiology, Concordia University, Montreal, QC Canada; 5 Alpert Medical School of Brown University & The Miriam Hospital, Providence, RI USA; 6 School of Medicine, University of Leeds, Leeds, United Kingdom; 7 Department of Human Nutrition, Foods and Exercise, Virginia Tech, Blacksburg, VA USA; 8 Department of Pediatrics, Center for Endocrinology, Diabetes and Metabolism, Children’s Hospital Los Angeles and Keck School of Medicine of University of Southern California, Los Angeles, CA USA; 9 Fit Minded Inc, Phoenix, AZ USA; 10 Charles Perkins Centre, The University of Sydney, Sydney, NSW Australia

**Keywords:** Obesity, Weight management

## Abstract

**Background:**

Weight stigma and weight discrimination refer to the negative attitudes and behaviours towards people with higher weight, because of their body size. Experiences of weight stigma in healthcare settings and within weight management interventions contribute to poor psychological and physical health and healthcare avoidance. This study aimed to develop a checklist for healthcare professionals and providers of weight management interventions to minimize weight stigmatisation, to the extent possible, in these interventions and settings.

**Methods:**

Based on existing literature on the contributors to weight stigma in healthcare, members of the Eating Disorders In Weight-Related Therapy (EDIT) Collaboration Weight Stigma Working Group drafted and refined the checklist items using an iterative process. The items were then pilot-tested by two reviewers. A 21-item checklist, organized into four domains: (1) planning and personnel; (2) intervention design and content; (3) outcomes and monitoring; and (4) additional components, was validated via an online survey. Healthcare professionals, researchers, and individuals with lived experience of higher weight evaluated the clarity, relevance, and importance of each item. The Content Validity Index (CVI), Content Validity Ratio (CVR), and participant feedback were used to refine the final version of the checklist.

**Results:**

Respondents (*n* = 28, 79% women, *M*_*age*_ = 44, *SD* = 10.6) completed the survey. Most items were rated as clear, and 20 out of 21 items were deemed relevant to the checklist’s aim (CVI range 0.61–1.0). Four items were rated as essential, with many remaining items rated as ‘important but not essential’. The final checklist consists of nine items across three sections: (A) essential elements of planning/design, (B) additional considerations, and (C) feedback on the service.

**Conclusion:**

The EDIT Weight Stigma Reduction Checklist has value in assisting providers to design, deliver, and implement weight management interventions to take a priori action to minimise weight stigma in practice.

## Introduction

Weight stigma refers to the negative attitudes, stereotypes, and behaviours towards people with higher weight, because of their body size [1]. Weight stigma is pervasive across settings including the media [2], education and employment [3, 4], and healthcare settings [5]. There is extensive evidence that weight stigma is associated with negative outcomes for people with higher weight. First, weight stigma prospectively predicts allostatic load [6] and mediates the prospective relationship between BMI and physiological dysregulation [7]. Second, meta-analytic evidence from a large body of research shows that weight stigma is negatively related to mental health [8] and healthy behaviours, like engaging in healthy eating and regular physical activity [9], and positively related to unhealthy eating behaviours and avoidance of exercise [9]. Finally, a recent systematic review of 242 articles found that experiencing weight stigma is related to binge eating, dietary restriction, bulimia symptoms and other eating disorder cognitions [10]. Therefore, it is essential to prevent and address weight stigma across settings.

Weight stigma is highly prevalent in healthcare settings. Healthcare professionals across disciplines and levels of training have both shown implicit and explicit weight stigma [for a review, see ref. [11]. Furthermore, evidence shows that this stigma manifests in differential clinical decision making in treatment of people of higher weight [12]. Individuals with higher weight report frequent experiences of weight discrimination (e.g., offensive weight-related comments, unsolicited weight-management advice, inadequate seating), in healthcare settings, e.g., refs. [13, 14]. This leads to distrust of healthcare professionals [15] and results in “doctor shopping” and healthcare avoidance [16]. While research has focused on the identification and assessment of explicit and implicit weight stigma among healthcare *trainees* and *professionals*, our research shows that behavioural weight management *interventions*, in and of themselves, are also a source of weight stigma. In a study aiming to understand the views of clinicians, researchers and consumers on factors that might contribute to eating disorder risk during weight management interventions [17], weight stigma was highlighted as a key factor. Some respondents suggested that interventions aiming to change weight are inherently stigmatising, and that weight stigma can be embedded in the design, delivery, and implementation of these interventions. For example, participants noted that use of inappropriate language, primary focus on weight, images as part of intervention materials, and the physical setting, such as chair size, are important considerations. The focus on weight, rather than on more “holistic” outcomes, also manifests in research on interventions. For example, a systematic review of interventions for uncomplicated obesity [18] found that only 37 out of 134 included studies reported on psychological/wellbeing outcomes in addition to reporting on weight loss (only 2 out of 11 studies reporting on lifestyle, and 7 out of 21 reporting on behavioural and psychological interventions). The prevalence of stigma in weight management interventions is particularly concerning given that behavioural weight management is considered a core component of obesity treatment, and adjunctive therapy to pharmacotherapy and bariatric surgery [19, 20]. Thus, it is vitally important that steps are taken to ensure that weight stigma is minimised in the design, delivery and implementation of interventions, and their evaluation. Despite this need, no tool currently exists to guide healthcare professionals and researchers on how to minimize weight stigma. This study aimed to develop and validate a checklist for researchers and healthcare professionals to minimise weight stigmatisation, to the extent possible, in the design and delivery of weight management interventions.

## Methods

### Study design

The development and validation of the checklist occurred over three phases: Phase 1, co-development and refinement of an initial checklist; Phase 2, validation of the checklist to assess the representativeness, comprehensibility and comprehensiveness of the items; and Phase 3, revision and refinement, based on the findings from Phase 2, to develop the final checklist.

### Ethics approval and consent to participate

Ethics approval was granted by the University of Sydney ethics review board (Project identifier: 2024/HE000246). Participants provided informed consent electronically through the survey platform.

### Phase 1: Co-development and refinement of initial checklist

The Eating Disorders In weight-related Therapy (EDIT) Collaboration [21] Weight Stigma Working Group was formed including lived experience experts, and researchers and clinicians with expertise in weight management interventions, eating disorders, and/or weight stigma. The Working Group co-developed the first version of the checklist, based on the available literature on contributors to weight stigma in healthcare and feedback received through a broader EDIT consultation process undertaken to identify contributors to eating disorder risk during weight management [17]. The initial development of items was informed by the EDIT Intervention deconstruction Framework [22], a comprehensive list of components of weight management interventions that influence eating disorder risk. Across each of the components, weight stigma research experts identified potential sources of stigma in this list, and considered how these could be mitigated, thereby generating the items. Items covered topics including staff weight bias minimisation training, using non-stigmatising language, selection of spaces, furniture and equipment that accommodate larger bodies safely and comfortably, setting and monitoring goals beyond weight loss, and addressing weight stigma internalisation. Items were refined through an iterative process with members of the working group.

Prior to validation, the survey was pilot tested with two reviewers in Qualtrics (one clinician-researcher and one person with lived experience). Reviewers were asked to provide feedback on individual items and to identify errors in form and presentation of the survey and use of the online system. Following pilot testing, the checklist was modified to include 21-items across four domains: (1) planning and personnel, (2) intervention design and content, (3) outcomes and monitoring, and (4) additional components. Items in this initial checklist can be found in Table 1.

**Table 1:** Clarity, content validity index, and content validity ratio for each item, with the corresponding total respondents (*n*) for each index.

Checklist item stems, by section	Clarity	*n*_*clarity*_	CVI	*n*_*CVI*_	CVR	*n*_*CVR*_	CVR + i
*Planning and personnel*							
1. Providers/clinicians receive training on weight stigma and how weight stigma may affect client interactions.	0.97	30	**0.90**	29	**0.93**	30	1.00
2. Providers/clinicians receive training on weight-related communication and using weight-related terminology preferences.	0.87	30	**0.93**	29	**0.67**	30	1.00
3. Person-first language is used in intervention materials (printed/distributed materials, online resources, apps, etc.).	0.90	30	**0.93**	29	**0.87**	30	0.93
4. Are considerations made to ensure that the equipment and/or built environment support diverse body sizes?	0.83	30	**1.00**	28	**0.93**	30	1.00
*Intervention design and content*							
5. Will mental health and wellbeing be assessed, and with appropriate support and/or referral provided if required?	0.76	29	0.76	29	0.10	29	0.93
6. Will the individual goals of the person be discussed in line with person-centred care?	0.68	28	**0.89**	27	0.29	28	0.85
7. Will the intervention include setting goals/targets with the person that consider aspects of health, other than weight?	0.86	29	**0.93**	29	**0.66**	29	0.93
8. Will access to long-term support be made available after the intervention?	0.76	29	0.61	28	0.17	29	0.51
*Outcomes and monitoring*							
9. Is there consideration for how people will be weighed, including asking for consent before weighing, offering blind weighing ensuring weighing takes place in a confidential space?	0.86	28	**0.96**	28	**0.86**	28	1.00
10. Are dimensions of health, other than weight, measured?	0.93	28	**0.96**	28	**0.50**	28	1.00
11. Are the measured dimensions of health, other than weight, discussed with the person (i.e., feedback on measures)?	0.86	28	**0.93**	28	**0.36**	28	0.85
*Additional components*							
12. Provide education on weight-focused communication skills (e.g., how to respond when others make weight-related comments) as part of the intervention.	0.71	28	**0.96**	28	**0.36**	28	0.92
13. Provide education or resources to a person’s social support networks (e.g., partner or parents/siblings/family unit) on weight stigma as part of the intervention.	0.96	28	**0.93**	28	-0.14	28	0.92
14. Address body image concerns as part of the intervention.	0.93	28	**0.86**	28	0.21	28	0.85
15. Address self-esteem as part of the intervention.	0.85	27	**0.85**	26	-0.14	28	0.92
16. Address internalised weight bias as part of the intervention.	0.82	28	**0.96**	27	**0.64**	28	0.92
17. Measure weight bias internalisation as an outcome.	0.82	28	**0.96**	27	0.29	28	0.92
18. Promote body compassion/acceptance as part of the intervention.	0.82	28	0.79	28	0.14	28	0.78
19. Provide strategies to increase resilience to public/structural weight stigma as part of the intervention.	0.86	28	**0.93**	28	0.29	28	0.78
20. Provide support/referral to address impacts of weight stigma (e.g., counselling, referrals for resultant mental health concerns).	0.89	28	**0.93**	28	0.21	28	0.92
21. Seek feedback on experiences of stigma during the intervention.	0.79	28	**0.85**	27	**0.36**	28	0.71

*Clarity* proportion of responses indicating question was clear, *CVI* Content Validity Index, *CVR* Content Validity Ratio, *CVR* + *i* Content validity ratio, calculated using the sum of “essential” and “important, but not essential” responses to the item.

Bolded numbers indicate criterion is met.

### Phase 2: Validation of the checklist

#### Participants

We recruited adults who belonged to one or more of the following three categories. First, clinicians or researchers who had conducted, and/or led the design or evaluation of, weight management interventions (behavioural, pharmacological or surgical). Second, people with lived experience of higher weight. Third, weight stigma researchers who had published at least one original research paper on weight stigma in the last 3 years.

#### Recruitment process

Members of the Weight Stigma Working Group who had not been involved in the development of the initial checklist were invited to participate via email. We also sent targeted invitations to weight stigma experts, and professionals delivering or designing weight management interventions. We aimed for a minimum sample size of 20 participants, with at least two participants from each target population (clinicians, researchers and lived experience). Recruitment occurred from 24/7/24 to 20/8/24.

#### Measures

##### Validation questionnaire

Validation questions aimed to assess the content validity of checklist items (i.e., extent to which the checklist items were deemed to be an adequate representation of the checklist goal). Participants were told that the aim of the checklist was “to assess the extent to which weight stigma is considered and minimised in weight management interventions”. For each item participants were asked to answer three questions: (a) is the question clear? (Yes, No), (b) is the question representative of the goal of the checklist? (Yes, No), and (c) is the question essential? (Essential, Important but not essential, Not essential). They were also given the opportunity to provide additional comments for each item and the checklist overall.

##### Demographics questions

To characterize the sample, participants were asked to provide information about their age, gender, country of residence, ethnicity, and professional background. Clinicians were asked to specify their discipline (e.g., psychologist, psychiatrist, nurse), their area of clinical expertise (e.g., eating disorders, obesity), the client age group they work with, and the number of years of experience in the area. Researchers were also asked to specify the relevant area(s) of research expertise, age group they work with, years of experience, and number of articles published in the relevant area of expertise. Lived experience experts were asked to specify whether their lived experience was of living with a higher weight and/or an eating disorder, whether they had lived experience of weight stigma in healthcare settings, and whether they had experience participating in a weight management intervention or receiving treatment for an eating disorder. Participants could indicate belonging to as many of the groups listed above as appropriate.

#### Procedure

The validation questionnaire was delivered via the online platform Qualtrics. Participants received a link to the survey, which landed on the participant information and consent form. Participants provided consent electronically, then proceeded to the validation questions, followed by demographic questions. The survey took ~20 min to complete.

#### Data analysis

For each checklist item, we calculated the proportion of responses that indicated that the item was clear. We then calculated the content validity index (CVI) as the proportion of ‘yes’ responses to the representativeness question. When values of CVI > 0.79, the item was deemed to be relevant; when 0.70 ≥ CVI ≤ 0.79, the item was deemed to need revisions; items were eliminated when CVI < 0.70 [23, 24].

Finally, we calculated the content validity ratio (CVR) for each item [25]. The CVR indicates whether an item is deemed to be essential. It is estimated with the formula CVR = (*Ne*–*N*/2)/(*N*/2), where *Ne* is the number of panellists identifying an item as “essential” and *N* is the total number of respondents. Higher CVR scores represent higher degree of essentiality. The number of respondents that need to agree that an item is essential, such that the level of agreement exceeds chance, for a sample of *n* = 28, is 19 (CVR = 0.357) [26]. We also calculated the CVR such that the “essential” and “important but not essential” responses were summed and entered for *Ne*; we called this index CVR+i. This was done to estimate of the extent to which low CVR values were due to people deeming that an item should be removed. Qualitative responses informed modifications to wording of individual items and checklist structure.

### Phase 3: Revision and refinement of the checklist

Data from the validation survey, together with free text comments were collated and discussed by the Working Group at an expert consensus meeting. Changes to the checklist were agreed upon after those discussions.

## Results

### Sample characteristics

Forty participants clicked on the link to the survey and provided consent, but only thirty answered the first question. Further, two participants dropped out of the survey before answering the demographic questions, which were presented last. Thus, we report demographics only for these 28 participants but include all data available for the validation questions.

Participants had *M*_*age*_ = 44 (*SD* = 10.6) years and 79% identified as women. Participants were from Canada [*n* = 12 (43%)], USA [*n* = 7 (25%)], Australia [*n* = 3 (11%)], UK/Great Britain/Northern Ireland [*n* = 3 (11%)], Belgium [*n* = 2 (7%)], Sweden [*n* = 1 (4%)]. The group included 25 researchers and 15 clinicians, including 12 reporting both professions. Participants reported expertise in working with children (age <10), adolescents (ages 10–18) and adults (age >18) and most reported working across overweight/obesity and eating disorders. Clinicians were clinical psychologists (*n* = 10), endocrinologists (*n* = 4) and one obstetrician. Researchers reported expertise across weight stigma (*n* = 21), weight management interventions (*n* = 20), and a range of eating disorders (*n* = 14). Most weight stigma researchers reported also working on research on weight management (*n* = 16) and/or eating disorders (*n* = 13).

Seven participants reported lived experience of higher weight (one clinician, three clinician/researchers, and three researchers). Of those with lived experience, four (57%) reported lived experience of BMI ≥ 40 kg/m^2^. Further, six (86%) reported lived experience of being the target of weight stigma in healthcare settings, and three (43%) reported lived experience of an eating disorder: atypical anorexia (*n* = 1) and binge eating disorder (*n* = 2). Participants with lived experience of higher weight reported previous experience with weight management treatment (*n* = 4), current experience with such treatment (*n* = 1), and never having received weight management treatment (*n* = 2).

### Validation findings

Table 1 displays the values for clarity, CVI and CVR for each item. The table shows most items were deemed to be clear (*M*_*clarity*_ = 0.84, *SD* = 0.08; Min = 0.67, Max = 0.96). Items 6 and 12 had clarity ratings of less than 0.75, suggesting a need for modification. Most of the comments noted that “person-centered care” (Item 6) would not be necessarily understood by everyone and would need to be defined/explained. For Item 12, comments indicated that it was not clear who was to receive the “education”.

The CVI indices in Table 1 show that all but three items (5, 8 and 18) met the criterion of relevance. Of these three, Item 8 (referring to providing long-term support after the intervention) was the only item for which the CVI value indicated the item should be discarded. Given this item also had a low clarity and low CVR, the item was removed. The CVI for the other two items (assessment of mental health with provision of referrals, and promotion of body compassion/acceptance) suggested the need for modification (see section on changes, below).

The CVR index presented less agreement on whether items were ‘essential’ for the checklist or ‘important, but not essential’. Table 1 shows that those items with low CVR had better scores when both “essential” and “important, but not essential” responses were included (CVR + i). For this reason, the comments provided for those items were considered by the working group when revising the items included in the list and the overall structure of the checklist, rather than completely discarding items.

### Modifications to the checklist

When revising individual items and the overall structure of the checklist, we combined the numerical analysis above and participants’ comments. The checklist was initially structured to align with the steps of intervention design and delivery. Based on the feedback received, the checklist was modified to include nine items across three sections (A) essential elements of planning/design; (B) additional considerations and (C) feedback on the service. This restructure was necessary as participants’ comments indicated that items across sections could be combined to reduce duplication. For example, *setting goals* beyond weight loss and *measuring* those goals should both be part of the same component of design of the intervention. The final version of the 9-item checklist is presented in Table 2.

**Table 2:** Final version of the 9-item checklist.

In the design and delivery of a weight management intervention, which of the following have been considered or provided:	Yes	N/A	Comments
(A) Essential elements of planning/ designing			
1. Providers/clinicians receive training about weight stigma and how weight stigma affects clinical consultations/interactions with clients. Training may be online or in-person and delivered by a recognised provider. A list of available resources for training can be found online at www.editcollaboration.com/resources: Training should include: a Debunking myths about weight regulation and understanding the complexity of obesity. b How assumptions about people of higher weight affect the clinician-client relationship. c Use of person-centered and non-stigmatising language. d The importance of choosing and using appropriate weight-related terminology with the client.			
2. Printed/distributed materials, online resources, apps, etc use non-stigmatising language (e.g., “person with obesity”, “person with higher weight”, “person living in a larger body”) and imagery. For example, imagery including people with a range of body sizes, remove images that exacerbate negative stereotypes, use available image libraries (see online resource guide). Resources to access non stigmatising images and information about language can be found online at www.editcollaboration.com/resources. Have materials co-designed by people with lived experience and clinicians to ensure they are appropriate.			
3. Equipment and/or built environments have been adapted to safely and comfortably support people with diverse body sizes, where possible for in-person interventions, including the following options:			
• Scales are positioned in a private space, and have a high maximum weight capacity			
• Examination gown sizes (have many different gown sizes available to accommodate diverse body sizes).			
• Appropriate assessment tools, with diverse sizes (e.g., blood pressure cuffs that accommodate diverse arm circumferences, long measuring tape, wide examination tables that can support high weights).			
• Seating (having diverse chair widths available in the intervention setting, with and without arm rests; seating should support a high maximum weight capacity to safely and comfortably support diverse body weights).			
• Built environment accessibility (e.g., shorter distances from the parking lot to the intervention setting, availability of a lift/elevator -especially if staircases are not designed to be safe and/or support people with mobility challenges, wide spaces within the waiting room area and doorways, suitable toilet facilities that safely and comfortably support higher weight capacities).			
• Exercise equipment (e.g., fitness equipment and benches have high maximum weight capacity and width to safely and comfortably support people with diverse body weights and sizes).			
4. Consider the context of weighing clients that includes (a) considering if weighing is necessary; (b) asking for consent before weighing, and (b) offering blind weighing (covering the number on the scale so it cannot be seen).			
5 Provide person-centred care in line with the client’s personal health goals. This may include discussing and setting goals beyond those related to weight loss, and measuring/monitoring progress related to these goals. These may include simple questions that invite self-reflection or can be assessed using validated measures. For example, goals discussed may include one or more of the following areas: (a) Quality of life [e.g., physical function (mobility, being able to perform tasks), self-esteem, sexual life, work, social relationships] (b) Cardiometabolic health markers (e.g., fitness, glucose levels, blood pressure, etc.) (c) Fruit and vegetable intake (d) Physical activity and sedentary behaviour (e) Self- or body- compassion (f) Other goals			
(B) Additional considerations			
6. Assess mental health (e.g., depression, anxiety, disordered eating, body image), determine cutoffs that necessitate mental health support and plan/establish appropriate referral pathways.			
7. Provide support to clients throughout the intervention to help them manage societal/external sources of weight stigma. This may include: (a) Training clients on communication skills that can help them with managing instances in which people in the client’s life want to engage in conversations about the person’s body weight (e.g., how to reframe conversations to focus on health instead of weight, how to say to others that their own weight is not up for discussion). (b) Providing guidance to a client’s social support networks (e.g., partner or parents/siblings/family unit, friends) on the negative effects of weight stigmatising language and treatment. (c) Providing clients with strategies to increase resilience and coping, so they are better able to deal with public/structural weight stigma as part of the intervention			
8. Address weight bias internalisation (WBI). This term refers to the extent to which a person adopts and endorses negative attitudes/beliefs about people with higher weight and, as a result, devalues and stigmatizes themselves because of their weight. This may include: (a) Explaining what WBI is and how it could affect their behaviours and their physical and mental health (b) Measuring WBI (before, during and after treatment) (c) Screening for high levels of WBI and develop a plan to address this as part of the intervention.			
(C) Feedback on service			
9. Seek feedback from clients/ participants on whether they experienced weight stigma during the intervention, as a mechanism for quality improvement of future services and interventions.			

EDIT weight stigma reduction checklist.

This checklist has been designed as a guide to minimise the risk of perpetuating weight stigma in weight management interventions.

This checklist includes: (A) a section with 5 items of “essential elements of planning/designing”, that should be implemented by all services, (B) a section with 3 items of ‘additional considerations’ of intervention elements intended to bring attention to outcomes other than weight loss alone and (C) a final item that recommends gathering feedback from participants on potential experiences of weight stigma throughout the intervention.

#### Essential elements of planning/designing section

This section brought together items with CVR above threshold from the original checklist’s first three sections. It combined these items so that the essential elements refer to (1) provider training, (2) intervention materials, (3) equipment and built environment, (4) processes for weighing, and (5) person-centred care. Participants indicated that provider/clinician training should include information about what weight stigma is and how it manifests in interactions between healthcare practitioners and clients. Participants noted that language used about weight/health is only one aspect of these interactions, noting that assumptions that practitioners make about the causes of obesity and about their clients’ behaviours (e.g., “patients lie about what they eat and how much they exercise”) can negatively affect those interactions, with downstream effects on patient engagement in healthcare. For this reason, the first item is more specific about the elements that should comprise training. In addition, and in response to participants comments, a link to a resource guide is now included in the checklist.

In response to participants’ comments, the original Item 3 (now Item 2), which referred to language use in materials, was expanded to include inclusive language *and* imagery, and links to resources are included in the guide. The wording of original Item 4 (now Item 3), which referred to “considerations” surrounding equipment and built environment was modified to use more directive language so that it is clear these adaptations must be implemented.

The item relating to weighing options for participants (formerly Item 9), was moved to this section, as it was rated as being essential to the planning/design stages of the intervention. The final item in the essential elements section combines content from Items 6, 7, 10 and 11. This item was generated in response to participants noting the need to define person-centred care, and that goal setting beyond weight loss, and the monitoring of these goals, constitute best practice and person-centred care. The checklist now makes explicit that goals other than weight loss *should* be discussed with clients and that steps should be taken to monitor and discuss achievement of these goals. Further, we added specific domains of relevant goals (e.g., quality of life, physical activity, etc).

#### Additional considerations section

This section of the checklist brought together items that originally referred to assessment of mental health, and provision of additional weight-stigma-related supports, both in relation to the interpersonal/structural and internalised stigma. Although most of the items included in this section had CVR indices below the threshold, they all were deemed to be either essential or important by most participants (CVR + i).

First, consideration of assessment of mental health (now Item 6) was deemed to be important because it is well known that weight stigma experiences mediate the relationship between higher weight and mental health. Thus, including assessments to identify potential mental health problems and establish appropriate referral pathways can support clients in mitigating the effects of their history of weight stigma. This item consolidated original items 5 and 20.

Second, providing clients with strategies that may help them navigate contexts and conversations where they face stigma, may increase the benefits of any intervention, by giving clients tools to stand up to, or to mitigate the effect of, weight stigma in their lives. The original items 12, 13 and 19 were all subsumed under Item 7, which incorporates participants’ wording suggestions to improve clarity.

The last item in this section focuses attention on another source of stigmatisation: weight bias internalisation (WBI), merging original items 14, 15, 16, 17 and 20. The item briefly defines WBI and suggests that providers discuss with clients what WBI is and how it can affect health behaviours and health outcomes. Further, it suggests monitoring of WBI and planning to address internalisation as part of the intervention.

#### Final considerations

The items that originally referred to body image and self-esteem were removed. This was done because participant comments noted that these issues would be subsumed under items referring to mental health, providing person-entered care and addressing WBI. Finally, the item referring to seeking feedback from clients was left as a standalone item, and expanded to emphasize the importance of self-reflection and quality improvement in healthcare as part of efforts to minimise stigma.

## Discussion

We present the development of a nine-item checklist designed to provide guidance to healthcare professionals, services and researchers to minimise weight stigma in weight management interventions. The checklist was developed with contributions from research and clinical experts with expertise in weight management and/or weight stigma research, and individuals with lived experience of higher weight. The checklist was evaluated by an independent multidisciplinary group of experts that included clinicians and researchers (some with lived experience of higher weight) with expertise in weight management, eating disorders and weight stigma.

The validation results indicated that all but two items were deemed clear by over 75% of participants, and the CVI values suggested that all but two items were considered relevant. However, according to the CVR indices only eleven items met the criterion of agreement beyond chance outlined by Ayre and Scally [26]. Of those, eight were brought together into a section of “Essential elements of planning/design”, which includes elements that *should* be incorporated before implementing an intervention. These include training providers about what stigma is and how to avoid it, using non-stigmatising language and imagery in any materials associated with the intervention, ensuring that the physical environment where the clients will interact with providers does not present barriers for them, and conducting weighing only with participant consent and on their terms, if at all. These four elements directly tackle aspects of healthcare provision that people of higher weight have found stigmatising and that are relevant to weight management settings [15]. Additionally, the final *essential* item of the checklist combined items that originally aimed to encourage person-centred care. The original decision to organise the items into planning, design, outcome monitoring and additional consideration sections, meant that goal setting and goal monitoring items appeared separately. Participants deemed this problematic, as goal setting and monitoring should not be considered independent from one another (goals set with the client should be monitored and discussed with them as the intervention progresses). For this reason, this final essential item in the list encourages practitioners to have conversations with their clients to set goals beyond weight loss and focus on other benefits that may arise from the health behaviour changes involved in weight management. The list of example goal-setting domains brings attention to aspects of a person’s life and health that may improve with changes in their health behaviours, even when weight loss is modest or null.

The second part of the list presents items described as “additional considerations”. These bring attention to other aspects of the clients’ life experience as a person with higher weight. First, this section suggests that mental health be assessed to facilitate referral to appropriate services if needed. This responds to extensive evidence that weight stigma experiences are related to mental health concerns, even after controlling for weight [8, 27]. Second, this section includes two items focused on providing clients with support to develop strategies to cope with a world where they face stigmatisation and where, as a result, they have internalised stigma. Suggested supports include providing training/resources to clients so they become more assertive in declining conversations about their weight and more resilient when facing structural stigma. Although we understand (and advocate) that it is society that needs to change and eliminate stigmatisation of people with higher weight, achieving this change is going to take time. Developing programs/interventions that empower individuals with strategies to identify and manage stigma when it happens may be helpful for their wellbeing. Additionally, this section suggests that healthcare professionals should provide resources/guidance to members of their clients’ support networks (family, romantic partners, friends, etc), to educate them about the negative effects of stigma, and debunk myths about obesity. This is important, as family-based weight stigmatisation is prevalent and is related to worse psychosocial outcomes [28].

Finally, the additional items refer to weight bias internalisation. Specifically, we suggest that healthcare professionals and researchers explain to clients what WBI is and how it affects an individual’s health behaviours, and physical and mental health outcomes. Further, we suggest that WBI be tackled, when possible, as part of the intervention (e.g., for an effective group-based internalised weight stigma intervention, delivered in combination with behavioural weight loss treatment see ref. [29]). Given that WBI is not only related to poorer wellbeing and body appreciation [10], but has also been shown to be related to engagement in avoidance of physical activity and unhealthy dietary behaviours [9], targeting WBI may facilitate engagement in behaviour change.

### Strengths and limitations

The final checklist was the result of input from a large panel with diverse clinical and/or research expertise in weight stigma, weight management and eating disorders, across a range of target age groups. The final checklist resulted from a structured validation process with strong empirical data. In particular, the ‘Essential Elements’ component includes items that were deemed to be clear and met our thresholds for validity (both with CVI and CVR index). The “Additional Considerations” component was built from items that were deemed to be clear and relevant (CVI) but did not meet the threshold to indicate they were deemed essential (although most were deemed important, albeit not essential (CVR+i)). Future research should evaluate the implementation and effectiveness of the checklist at reducing weight stigma and its consequences in clinical intervention settings.

Although the expert panel was large enough to assess content validity, given the selected CVI threshold [30], and their expertise was diverse, all participants were from developed countries. Further, future research may consider assessing the content validity of the tool (or a modified version of it) with experts from different cultural contexts and background.

An important issue to address is whether weight management interventions can aim to minimise stigma or whether this is a contradiction of terms. This issue was raised by at least one participant in this study, and in our prior research [17]. The argument is that an intervention that aims to induce weight loss, cannot be non-stigmatising because its very aim is to eliminate/reduce the characteristic that is the source of weight stigma (i.e., higher body weight). This is a valid point. However, we believe people continue to seek and engage in weight loss interventions, and their right to make that choice should be respected. As long as this is the case, it is important to ensure that these interventions minimise stigmatisation of their clients by (a) educating the healthcare professionals and researchers that design and deliver these types of interventions, (b) ensuring that the physical and human environments that people of higher weight encounter in these settings are less threatening and (c) extending the focus beyond weight, to other positive outcomes of health behaviour change. This checklist might encourage healthcare professionals and researchers to reflect and consider other aspects of their clients’ experiences. We hope that the changes that may be brought about by the adoption of this checklist, result in improved client outcomes. This is based on the understanding that weight stigma experiences are known to be related to poor mental health [8], increased healthcare avoidance [16], and lower engagement in healthy eating and exercise behaviours [9, 10]. Thus, reduction of stigma is expected to be associated with a reduction in these negative outcomes. This may be the focus of future research.

## Conclusion

The EDIT Weight Stigma Reduction Checklist aims to encourage healthcare professionals, providers and researchers of weight management interventions to minimise weight stigma in their practice. It does so by providing a list of items that should be incorporated in the planning and design of interventions. The checklist includes elements that many healthcare professionals consider minimal standards of best clinical practice. We agree that this should be the case. The checklist provides an opportunity to explicitly state and draw attention to the importance of considering all these elements when designing and implementing weight management interventions. Further, the checklist can also be used as a quality improvement tool to evaluate and modify existing interventions/services. Finally, we hope that this checklist encourages healthcare professionals and researchers working in weight management to consider the steps that they can take to reduce weight stigma in their interactions with their clients, and to empower them to manage the stigma they face in the world.

## Data Availability

No additional datasets were generated or analysed beyond what is reported in this article. All study data are included in aggregate within the published manuscript.
